# The diverse and unanticipated roles of histone deacetylase 9 in coordinating plant development and environmental acclimation

**DOI:** 10.1093/jxb/eraa335

**Published:** 2020-07-20

**Authors:** Peter G H de Rooij, Giorgio Perrella, Eirini Kaiserli, Martijn van Zanten

**Affiliations:** 1 Molecular Plant Physiology, Institute of Environmental Biology, Utrecht University, Padualaan, CH Utrecht, The Netherlands; 2 Institute of Molecular, Cell & Systems Biology, College of Medical, Veterinary and Life Sciences, University of Glasgow, Glasgow, UK; 3 ENEA - Trisaia Research Centre 75026, Rotondella (Matera), Italy; 4 University of Birmingham, UK

**Keywords:** ABA INSENSITIVE 4 (ABI4), Arabidopsis, EARLY FLOWERING 3 (ELF3), gene regulation, HDA9, histone deacetylase 9, HIGH EXPRESSION OF OSMOTICALLY RESPONSIVE GENES 15 (HOS15), ELONGATED HYPOCOTYL 5 (HY5), POWERDRESS (PWR), WRKY53

## Abstract

Plants tightly control gene transcription to adapt to environmental conditions and steer growth and development. Different types of epigenetic modifications are instrumental in these processes. In recent years, an important role for the chromatin-modifying RPD3/HDA1 class I HDAC HISTONE DEACETYLASE 9 (HDA9) emerged in the regulation of a multitude of plant traits and responses. HDACs are widely considered transcriptional repressors and are typically part of multiprotein complexes containing co-repressors, DNA, and histone-binding proteins. By catalyzing the removal of acetyl groups from lysine residues of histone protein tails, HDA9 negatively controls gene expression in many cases, in concert with interacting proteins such as POWERDRESS (PWR), HIGH EXPRESSION OF OSMOTICALLY RESPONSIVE GENES 15 (HOS15), WRKY53, ELONGATED HYPOCOTYL 5 (HY5), ABA INSENSITIVE 4 (ABI4), and EARLY FLOWERING 3 (ELF3). However, HDA9 activity has also been directly linked to transcriptional activation. In addition, following the recent breakthrough discovery of mutual negative feedback regulation between HDA9 and its interacting WRKY-domain transcription factor WRKY53, swift progress in gaining understanding of the biology of HDA9 is expected. In this review, we summarize knowledge on this intriguing versatile—and long under-rated—protein and propose novel leads to further unravel HDA9-governed molecular networks underlying plant development and environmental biology.

## Teaser

We discuss the contribution of the versatile and atypical plant histone deacetylase, HDA9, to plant growth, development, and acclimation to the environment, and propose novel leads for future research.

## Introduction

Eukaryotic DNA is orderly and densely packed into higher order structures, called chromatin. The first level of chromatin compaction comprises a histone protein octamer that wraps ~147 bp (Luger *et al.*, 1997; Rosa and Shaw, 2013). This basal protein–DNA unit, called a nucleosome, contains a tetramer of two dimers consisting of four core histone (H) proteins each: H2A/H2B and H3/H4. Besides the four canonical histones, various histone variants exist with different physical properties and biological functions (Henikoff and Smith, 2015; Talbert and Henikoff, 2017). Histone proteins contain unstructured N-terminal tails that extrude from the nucleosomes and are prone to post-translational epigenetic modifications such as acetylation, methylation, SUMOylation, ubiquitination, and phosphorylation (Berger, 2007; Rosa and Shaw, 2013; Liu *et al.*, 2014). Such epigenetic modifications regulate the accessibility of DNA to binding proteins, such as transcription factors and DNA polymerases, by modulating the electrostatic interactions between the histones and DNA molecule (Bowman and Poirier, 2015).

Histone acetylation is a dynamic and versatile epigenetic mark that occurs at lysine (K) residues on the histone tails and causes histones to shift from a positive to a neutral charge, thereby typically allowing for a transcriptionally prone, decondensed chromatin environment. Histone acetyltransferases (HATs) catalyze the deposition of acetyl groups, whereas histone deacetylases (HDACs) remove these marks (Pandey *et al.*, 2002; Liu *et al.*, 2014; Chen *et al.*, 2020). Hence, HDACs are associated with SWI-INDEPENDENT3 (SIN3)-like co-repressors and are often—but not exclusively—associated with silenced genes (Li *et al.*, 2002; Tian *et al.*, 2005; Alinsug *et al.*, 2009). Other factors in HDAC multiprotein co-repressor complexes typically are DNA-binding factors, chromatin-modifying enzymes, and several other structural and regulatory proteins (Grzenda *et al.*, 2009; Perrella *et al.*, 2013, 2016; Liu *et al.*, 2014). Together, HDAC multiprotein complexes orchestrate enzymatic activity, cofactor recruitment, substrate binding, and genomic targeting.

In *Arabidopsis thaliana*, there are 18 proteins recognized as HDACs that are categorized into three families: the Reduced Potassium Dependence3 (RPD3/HDA1-like) family, the plant-specific HD2-type family, and the NAD-dependent Silent Information Regulator (SIR) family. These families contain twelve, four, and two members, respectively. The RPD3/HDA1-like family is subdivided into three classes (I–III) based on sequence similarity (Pandey *et al.*, 2002; Hollender and Liu, 2008; Alinsug *et al.*, 2009). HDACs exert diverse functions in plants. For a detailed overview of HDACs, we refer the reader to Liu *et al.* (2014) and Chen *et al.* (2020).

In recent years, the RPD3/HDA1 class I HDAC HISTONE DEACETYLASE 9 (HDA9) has gained increasing attention. Phylogenetic analyses indicate that HDA9 is homologous to the functional HDACs: HDA6, HDA7, and HDA19 (Pandey *et al.*, 2002; Hollender and Liu, 2008; Alinsug *et al.*, 2009). In addition, *HDA9* is closely related to *HDA10* and *HDA17*, which are physically located next to *HDA9* on the genome. These pseudogenes lack a catalytic HDAC domain and probably originated from a HDA9 duplication and genomic rearrangement event (Pandey *et al.*, 2002; Alinsug *et al.*, 2009).

Unlike other functional plant HDACs, HDA9 contains a BH3-only pro-apoptotic (BAD) domain (Alinsug *et al.*, 2009), that allows for interaction with 14-3-3 proteins that are associated with a multitude of signaling proteins and have a role in hormone, kinase, phosphatase, and transmembrane receptor signaling pathways (Jaspert *et al.*, 2011; Camoni *et al.*, 2018).

*HDA9* expression is observed in several Arabidopsis organs and tissues across developmental stages, which suggests that HDA9 functions throughout the plant’s life cycle (Van Zanten *et al.*, 2014; Kang *et al.*, 2015; Suzuki *et al.*, 2018; Mayer *et al.*, 2019; van der Woude *et al.*, 2019) (Table 1). In germinating seedlings, *HDA9* is mainly present in below-ground parts and the root–hypocotyl junction (Van Zanten *et al.*, 2014; van der Woude *et al.*, 2019), and the gene becomes more ubiquitously expressed later in development (Hollender and Liu, 2008; Kang *et al.*, 2015; Mayer *et al.*, 2019). Accordingly, the *Brassica juncea HDA9* homolog (*BjuHDA9*) is ubiquitously detected throughout the plant and particularly in floral tissues (Yan *et al.*, 2018).

**Table 1.** Confirmed *HDA9* expression domains across plant developmental stages and their corresponding literature references

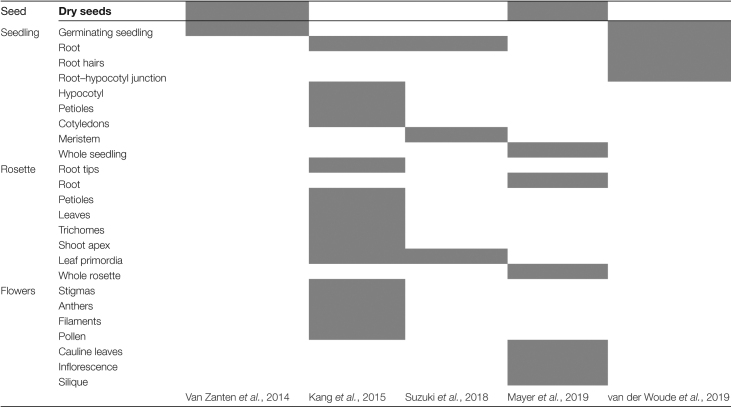

HDA9 substrates include H3K9Ac, H3K14Ac, H3K18Ac, H3K27Ac, H3K36Ac, and H3K56Ac (Kim *et al.*, 2013; Van Zanten *et al.*, 2014; Kang *et al.*, 2015; Chen *et al.*, 2016; Kim *et al.*, 2016; Mayer *et al.*, 2019; Park *et al.*, 2019; van der Woude *et al.*, 2019; Yang *et al.*, 2020; Zeng *et al.*, 2020; Zheng *et al.*, 2020), but not H4 or H2A lysines (Kim *et al.*, 2016; Mayer *et al.*, 2019). In addition, *hda9* mutants display altered H3K9Me1, H3K9Me2, H3K27Me1, H3K27Me2, and H3K36Me2 levels (Chen *et al.*, 2016; Kim *et al.*, 2016; Mayer *et al.*, 2019; Zeng *et al.*, 2020). How HDA9 affects histone methylation is unknown, but HDA9 is likely to play a facilitating role, as HDA9-mediated H3K27 deacetylation is required for Polycomb Repressive Complex 2 (PRC2)-mediated H3K27me3 (Zeng *et al.*, 2020). Furthermore, accumulation of miRNAs (miR157, miR162, and miR172) was impaired in the *hda9* mutant background, suggesting a possible role for HDA9 in the regulation of miRNA production (Kim *et al.*, 2009).

**Fig. 1.**
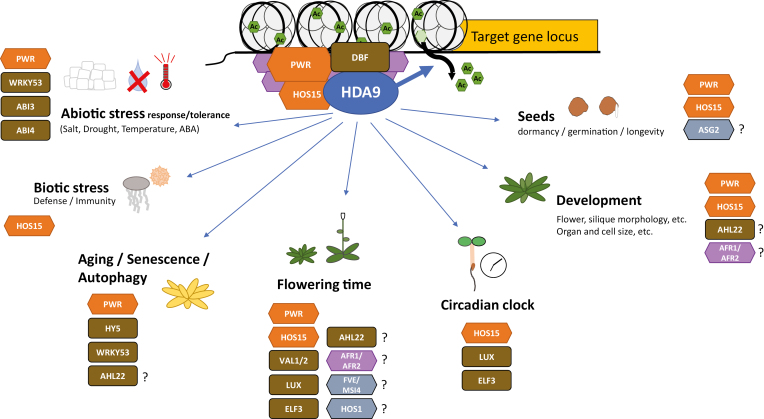
Schematic representation of the HDA9–PWR–HOS15 core histone deacetylase complex and their roles in plant development and responses to the environment. The catalytic HDAC HDA9 (blue oval), together with its core complex components PWR and HOS15 (orange elongated hexagons) and other structural components (purple hexagon), such as AFR1/AFR2, facilitate the de-acetylation (green hexagons) of histones in nucleosome complexes (gray circles), around which two turns of DNA are wrapped (black lines). This affects chromatin accessibility for regulatory proteins and the transcription machinery, and thereby controls the expression of its target genes (yellow box). The HDA9–core histone deacetylase complex is targeted to DNA promoter elements by DNA-binding factors (DBFs; brown boxes), that includes transcription factors such as WRKY53, HY5, ELF3, ABI3, and ABI4. Other known HDA9 partners are the DNA-binding proteins AHL22, VAL1, and VAL2, as well as ASG2, FVE/MSI4, and HOS1 (gray hexagons). The HDA9–PWR–HOS15 complex regulates diverse processes throughout the plant’s life cycle as well as responses and tolerance to the indicated biotic and abiotic stresses. The diverse HDA9-mediated processes and responses rely on different DNA-binding and other proteins (known factors are depicted in association with the mentioned process/response).

In general, HDA9 targets histones positioned close to transcriptional start sites of actively transcribed genes at euchromatic regions (Fig. 1) (Chen *et al.*, 2016; Kim *et al.*, 2016; Mayer *et al.*, 2019; Yang *et al.*, 2019). Consistent with its role in transcriptional regulation, the association of HDA9 with genomic targets correlates well with mRNA expression levels (Chen *et al.*, 2016; Kim *et al.*, 2016; Mayer *et al.*, 2019; Yang *et al.*, 2019; Baek *et al.*, 2020).

Despite the fact that some HDACs target non-histone protein substrates (Hartl *et al.*, 2017; Zheng *et al.*, 2020), for a long time there was no evidence suggesting that HDA9 can deacetylate proteins other than histone H3, even though HDA9 has been detected in both the cytoplasm and the nucleus (Kang *et al.*, 2015; Ducos *et al.*, 2017; Suzuki *et al.*, 2018; Mayer *et al.*, 2019; Yang *et al.*, 2019). A recent study, however, demonstrated that HDA9 can remove acetyl groups and thereby negatively regulates the transcriptional activity of its interacting transcription factor protein WRKY53 (Zheng *et al.*, 2020). Pharmacological evidence showed that HDA9 is prone to proteasomal regulation (Mayer *et al.*, 2019) and it has been suggested that HDA9 may be associated with a CUL4-based E3 ligase (Park *et al.*, 2019).

On the phenotypic level (Fig. 1), HDA9 regulates diverse traits including seed dormancy (Van Zanten *et al.*, 2014; Baek *et al.*, 2020), flowering time (Kim *et al.*, 2013; Kang *et al.*, 2015; Kim *et al.*, 2016; Mayer *et al.*, 2019; Park *et al.*, 2019; van der Woude *et al.*, 2019; Zeng *et al.*, 2020), leaf senescence (Chen *et al.*, 2016; Mayer *et al.*, 2019; Yang *et al.*, 2020), cellular differentiation (Lee *et al.*, 2016), cell proliferation (Suzuki *et al.*, 2018), suppression of stem cuticular wax crystal accumulation (Wang *et al.*, 2018), flower opening, petal and sepal attachment to the receptacles (Kang *et al.*, 2015), and several other developmental and physiological phenotypes (Fig. 1; Table 2). Moreover, HDA9 mediates responses to environmental signals such as salt, drought (Zheng *et al.*, 2016; Baek *et al.*, 2020; Khan *et al.*, 2020; Zheng *et al.*, 2020), and warm temperatures (Tasset *et al.*, 2018; Shen *et al.*, 2019; van der Woude *et al.*, 2019).

**Table 2.** Confirmed HDA9-mediated processes and responses to environmental stresses and their corresponding literature references

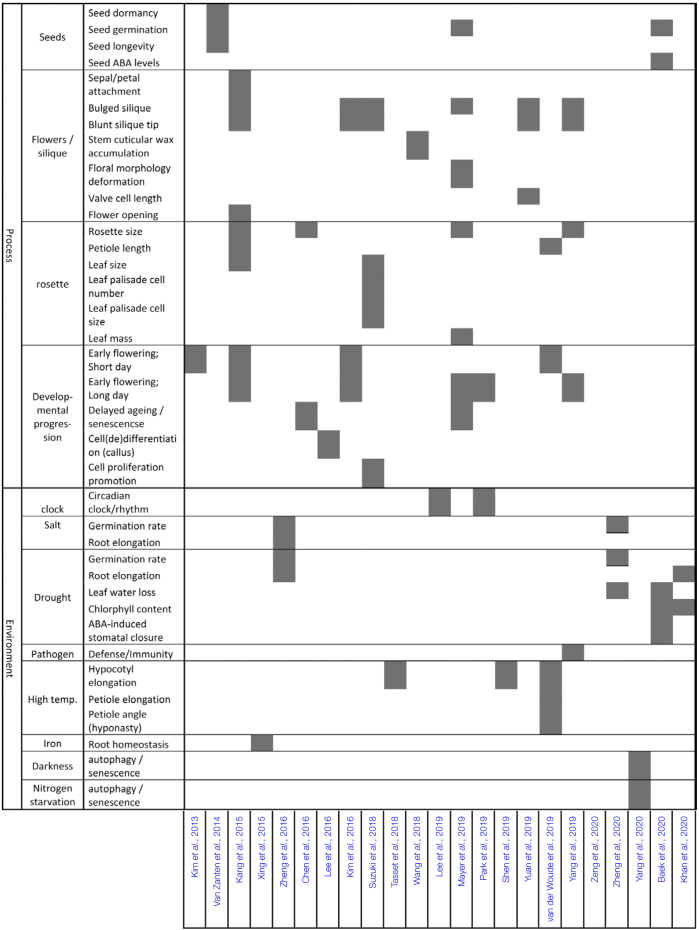

In this review, we report in detail the intriguing findings on the versatile role of the pleiotropic HDA9 chromatin-modifying protein (Fig. 1) and discuss possible future directions required to further unravel the function and regulation of HDA9-governed molecular networks.

## HDA9-interacting proteins; the HDA9–HOS15–PWR core HDAC complex

The SANT (Swi3, Ada2, N-Cor, TFIIIB) domain-containing protein POWERDRESS (PWR) was identified by an immuno-purification approach as a high-confident HDA9-interacting protein (Chen *et al.*, 2016) (Fig. 1; Table 3). In addition, HDA9 was identified in a screen for *hdac* mutants with early flowering and bulged silique phenotypes similar to *pwr* mutants (Yumul *et al.*, 2013; Kim *et al.*, 2016). Consistent with the proposed role for PWR in HDAC multiprotein complexes, a histone H3 hyperacetylation phenotype was observed in *pwr* mutants, and *pwr*-hyperacetylated sites significantly overlapped with those found in the *hda9* mutant background (Chen *et al.*, 2016; Kim *et al.*, 2016; Mayer *et al.*, 2019). Furthermore, the WD40-repeat protein HIGH EXPRESSION OF OSMOTICALLY RESPONSIVE GENE 15 (HOS15) was shown to interact with both HDA9 and PWR (Fig. 1; Table 3) (Park *et al.*, 2018*a*, *b*; Suzuki *et al.*, 2018; Mayer *et al.*, 2019; Park *et al.*, 2019; Yang *et al.*, 2019), and the *hos15* mutant displayed histone hyperacetylation and methylation changes similar to *hda9* and *pwr* mutants (Suzuki *et al.*, 2018; Mayer *et al.*, 2019; Yang *et al.*, 2019). Moreover, HDA9 chromatin binding was reduced in *hos15* (Chen *et al.*, 2016) and *pwr* mutants (Kim *et al.*, 2016), suggesting that PWR and HOS15 are required for HDA9 genome targeting.

**Table 3.** Confirmed HDA9-interacting proteins

Interacting protein	Reference	Technique(s) used for interaction study	Target gene identification/ confirmation method(s)^*a*^
**WRKY53**	Zheng, 2020	Y2H, Co-IP, BiFC	qRT-PCR, ChIP-PCR, transient expression assays
**PWR, WRKY53, AHL22**	Chen, 2016	Co-IP, *in vitro* IP	qRT-PCR, RNA-seq, ChIP-PCR, ChIP-seq
**PWR**	Kim, 2016	Co-IP, Y2H	qRT-PCR, RNA-seq, ChIP-PCR, ChIP-seq
**HOS15, PWR**	Suzuki, 2018	Y2H	qRT-PCR
**HOS15, PWR**	Mayer 2019	IP-MS, IP, BiFC	qRT-PCR, RNA-seq, ChIP-PCR, ChIP-seq
**HOS15**	Yang, 2019	Co-IP	qRT-PCR, RNA-seq, ChIP-PCR
**HOS15**	Park 2018*a*	Split-LUC	qRT-PCR, ChIP-PCR
**HOS15**	Park 2018*b*	IP-MS, Co-IP, Y2H, LCI	NA
**HOS15, ELF3, LUX**	Park 2019	Co-IP^*b*^	qRT-PCR, RNA-seq, ChIP-PCR
**ELF3**	Lee, 2019	Y2H, Co-IP, BiFC	qRT-PCR, ChIP-PCR, transient expression assays.
**AHL22**	Yun, 2012	BiFC, *in vitro* pulldown	qRT-PCR, ChIP-PCR, EMSA, MAR binding assay
**AFR1, AFR2**	Gu, 2013	Y2H	qRT-PCR, ChIP-PCR
**HOS1, FVE/MSI4**	Jung, 2013	Y2H	qRT-PCR, ChIP-PCR
**CYP1-1. HDA6, HDA19** ^***c***^	Zheng, 2016	Y2H	qRT-PCR, RNA-seq, ChIP-PCR
**ASG2**	Ducos, 2017	BiFC	NA
**VAL1, VAL2**	Zeng, 2020	Co-IP, Y2H	qRT-PCR, ChIP-PCR
**HY5**	Yang, 2020	Co-IP, BiFC	qRT-PCR, ChIP-PCR, dual-luciferase reporter assay
**ABI4**	Baek, 2020	Co-IP, Y2H	qRT-PCR, ChIP-PCR
**ABI4, ABI3**	Khan, 2020	Co-IP, Y2H	qRT-PCR, ChIP-PCR

^*a*^ The indicated techniques were used to identify target genes of either HDA9 and/or of the specified HDA9-interacting protein.

^*b*^ Co-IP data by Park *et al.* (2019) suggest HDA9–LUX interaction, but a yeast two-hybrid assay did not confirm this interaction (Lee *et al.*, 2019).

^*c*^ These proposed interactions (yeast two-hybrid-based) should be considered with care, as Yuan *et al.* (2019) did not detect interaction between HDA9 and HDA6.

Abbreviations; Y2H, yeast two-hybrid; Co-IP, co-immunoprecipitation; IP-MS, immunoprecipitation followed by MS; BiFC, bimolecular fluorescence complementation; LCI, luciferase complementation imaging, qRT-PCR, quantitative real-time PCR; ChIP-PCR, chromatin immunoprecipitation-PCR; ChIP-seq, ChIP sequencing; RNA-seq, RNA sequencing (whole-transcriptome sequencing); MAR, matrix-attachment region; NA, not applicable.

 Several *hda9* mutant phenotypes, including altered leaf size, leaf palisade cell number and palisade cell size (Suzuki *et al.*, 2018), and other traits further discussed below, are equally affected in *hos15* (and *pwr*) single mutants and, to the best of our knowledge, no clear additive effects were observed in higher order mutants for any of the tested phenotypes. Moreover, *HDA9*, *PWR*, and *HOS15* are co-expressed in different tissues (Mayer *et al.*, 2019) and *hda9*, *hos15*, and *pwr* mutant transcriptomes exhibit a large overlap (Chen *et al.*, 2016; Mayer *et al.*, 2019). In fact, nucleocytoplasmic fractionation assays demonstrated that PWR and HOS15 are required for HDA9 accumulation in the nucleus, and *pwr* and *hos15* mutants show significantly reduced nuclear HDA9 levels (Chen *et al.*, 2016; Mayer *et al.*, 2019). However, the mutant transcriptomes of *pwr* and *hos15* suggest that both display HDA9-independent effects on gene regulation, possibly by interacting with other HDAC transcriptional co-repressors. Indeed, unlike HDA9 and PWR, HOS15 also targets acetylated H4 (Zhu *et al.*, 2008).

Taken together, HDA9–PWR–HOS15 form a core HDAC complex to control gene transcription (Fig. 1). In addition, HDA9 physically interacts with the DNA-binding AT-HOOK MOTIF-CONTAINING 22 (AHL22) protein (Yun *et al.*, 2012; Chen *et al.*, 2016) and with AP30 FUNCTION-RELATED 1 (AFR1) and AFR2, being the plant relatives of yeast SAP30 FUNCTION-RELATED 1, a Sin3-associated structural component of HDAC complexes (Gu *et al.*, 2013) (Table 3). Up to now, the contribution of AHL22 and AFR1/AFR2 to HDA9-mediated phenotypes is poorly understood. However, *AHL22* overexpression results in short and stunted siliques and compact plants (Yun *et al.*, 2012), similar to *pwr* and *hda9* mutants. However, unlike *pwr* and *hda9* (Kim *et al.*, 2013; Yumul *et al.*, 2013; Kang *et al.*, 2015; Kim *et al.*, 2016; Mayer *et al.*, 2019; Park *et al.*, 2019; van der Woude *et al.*, 2019; Yang *et al.*, 2019; Zeng *et al.*, 2020), *AHL22* overexpression leads to delayed flowering (Yun *et al.*, 2012). Furthermore, *afr1* and *afr2* mutants exhibit elongated petioles and an open rosette structure, which is in contrast to the stunted *hda9* mutant phenotype (Gu *et al.*, 2013), whereas similarly to *hda9* and *pwr*, the *afr* mutants exhibit early flowering (Gu *et al.*, 2013). Additional proteins shown to interact with HDA9 include ASG2 (Ducos *et al.*, 2017), EARLY FLOWERING3 (ELF3), and possibly LUX ARRHYTHMO (LUX) (Lee *et al.*, 2019; Park *et al.*, 2019), VP1/ABI3-LIKE 1 (VAL1) and VAL2 (Zeng *et al.*, 2020), ELONGATED HYPOCOTYL 5 (HY5) (Yang *et al.*, 2020), and ABA INSENSITIVE4 (ABI4) and ABI3 (Baek *et al.*, 2020, Khan *et al.*, 2020). These interactions are discussed below (Fig. 1; Table 3). The biological function of the indicated interaction between HDA9, HOS1, and FVE/ MULTICOPY SUPPRESSOR OF IRA1 4 (MSI) (Jung *et al.*, 2013) requires further investigation (Table 3).

Interestingly, similar to the *hda9* mutant phenotypes, mutants in histone deacetylase complex 1 (HDC1), a factor that interacts with HDACs and quantitatively determines histone acetylation levels, exhibited short petioles and a compact stature (Perrella *et al.*, 2013, 2016). This suggests that HDC1 may also be part of the HDA9–PWR–HOS15 multiprotein complex. However, a possible direct interaction between HDC1 and HDA9 remains to be established. Notably, to the best of our knowledge, the HDA9-interacting proteins so far identified are fundamentally different from other HDACs studied. In particular, co-immunoprecipitation (Co-IP) using HDA6 or HDA19 as baits revealed mainly interactions with the conserved subunits of the RPD3-containing HDAC complex, including SIN3-like co-repressor proteins (SNL1–SNL6) and MSI1, and with each other (Perrella *et al.*, 2013; Mehdi *et al.*, 2016; Ning *et al.*, 2019). This could indicate that the HDAC complex containing HDA9 may be fundamentally divergent from related canonical HDACs.

The transcription factors WRKY53 (Chen *et al.*, 2016; Zheng *et al.*, 2020), ABI4, ABI3 (Baek *et al.*, 2020, Khan *et al.*, 2020), and HY5 (Yang *et al.*, 2020), the epigenome readers VP1/ABI3-LIKE 1 (VAL1) and VAL2 (Zeng *et al.*, 2020), the circadian clock Evening Complex (EC) transcription factor(s) ELF3 and possibly LUX (Lee *et al.*, 2019; Park *et al.*, 2019), and AT-hook motif-containing protein AHL22 (Yun *et al.*, 2012) are currently the only confirmed HDA9-interacting proteins with DNA binding capacity (Fig. 1). In particular, the molecular mechanism of the HDA9–WRKY53 interaction is now understood in detail (Zheng *et al.*, 2020). Despite the limited number of confirmed HDA9 interactors, it is likely that HDA9 associates directly—or as part of a bigger HDAC multiprotein complex—with many more yet to be discovered DNA-binding factors.

## The role of HDA9 in circadian clock regulation

Coordinated plant growth and development depend on tight regulation by the circadian clock. Circadian rhythms are entrained by environmental cues such as daylength and ambient temperature, and regulate vital processes such as metabolism, energy homeostasis, plant growth, stomatal closure, positional movement of leaves, and flowering initiation (Jouve *et al.*, 1998; Dowson-Day and Millar, 1999; McClung, 2006; Park *et al.*, 2019). At the core of the complex circadian clock regulation are multiple interlocking transcriptional feedback loops that regulate the clock’s output across a day/night cycle. The so-called central oscillator consists, among other factors, of two morning-expressed MYB transcription factors, LATE ELONGATED HYPOCOTYL (LHY) and CIRCADIAN CLOCK-ASSOCIATED 1 (CCA1), and the evening-expressed TIMING OF CAB EXPRESSION 1 (TOC1) (Gendron *et al.*, 2012) (also referred to as PSEUDO RESPONSE REGULATOR 1 or PRR1), as well as other PRR family members such as PPR5, PPR7, and PPR9 (Nakamichi *et al.*, 2010; Chow *et al.*, 2012), GIGANTEA (GI), and the EC factors LUX, ELF3, and ELF4 (Ezer *et al.*, 2017; McClung, 2019).

Over a third of Arabidopsis gene transcripts are controlled by the circadian clock (Michael and McClung, 2003; Kim *et al.*, 2017), and rhythmic chromatin modifications have been associated with Arabidopsis circadian clock regulation (Farinas and Mas, 2011; Malapeira *et al.*, 2012; Hung *et al.*, 2018). The activity of CCA1, together with the MYB transcription factor REVEILLE8 (RVE8) for instance, causes differential H3 acetylated states at the *TOC1* promoter region. At dawn, CCA1 represses chromatin accessibility via the recruitment of HDACs or repression of HATs (Perales and Más, 2007). During the daytime, CCA1 is antagonized by RVE8, correlating with H3 acetylation (Farinas and Mas, 2011; Malapeira *et al.*, 2012; Hung *et al.*, 2018), and rhythmic changes in histone marks are closely associated with clock activity (Perales and Más, 2007; Farinas and Mas, 2011; Lee *et al.*, 2019).

A recent study demonstrated that expression of the circadian clock genes *CCR2*, *CAB2*, *CCA1*, and *TOC1* displays signs of period shortening and advanced rhythmic phase in the *hda9* mutant background (Lee *et al.*, 2019). However, *HDA9* expression itself did not show a significant circadian oscillation in wild-type plants. Subsequent analysis demonstrated that HDA9 is recruited to the *TOC1* promoter region, thereby promoting H3 deacetylation. This resulted in *TOC1* repression after its peak expression during the night (Lee *et al.*, 2019).

Furthermore, it was recently found that HDA9 interacts with ELF3 when in complex with LUX (Lee *et al.*, 2019; Park *et al.*, 2019) (Table 3). Whether HDA9 directly interacts with LUX is not yet clear. Co-IP data by Park *et al.* (2019) would suggest so. Yet, a yeast two-hybrid assay did not confirm a direct interaction between the two proteins (Lee *et al.*, 2019). Nevertheless, *TOC1* repression is mediated by HDA9 via a direct and rhythmic interaction with ELF3 (Lee *et al.*, 2019). Indeed, HDA9-dependent deacetylation and HDA9 association with the *TOC1* promoter was impaired in *elf3* mutants, comparable with *hda9* mutants. Similarly, the HOS15–HDA9–EC complex dampens the rhythmic expression of *GI*, by mediating the deacetylation of *GI-*associated histone proteins, mainly in the late afternoon (Park *et al.*, 2019). Moreover, it was shown that HOS15–HDA9 is targeted to the *GI* locus by LUX and ELF3 and that this is necessary for the deacetylation of H3 at the *GI* promoter to repress flowering (Park *et al.*, 2019).

## HDA9 control of flowering time

Flowering time is tightly regulated by several endogenous developmental cues and environmental variables such as temperature and photoperiod (Cho *et al.*, 2017). Several studies have reported an intrinsic role for HDA9 in flowering time control.

Mutations in HDA9 lead to a mild early flowering phenotype under otherwise non-inductive short-day (SD) photoperiod conditions, seemingly independent of the CONSTANS/SUPPRESSOR OF OVEREXPRESSION OF CO1 (CO/SOC1) pathway (Kim *et al.*, 2013; Kang *et al.*, 2015; Kim *et al.*, 2016; van der Woude *et al.*, 2019). Subsequent analysis revealed that *hda9* mutants show increased expression levels of the floral activator *AGAMOUS-LIKE19* (*AGL19*) in SDs, which is accompanied by increased H3K9Ac and H3K27Ac levels at the *AGL19* chromatin. Subsequent ChIP experiments indicated that HDA9 is indeed capable of binding to the *AGL19* locus and directly affects *AGL19* transcription by mediating deacetylation, thereby repressing flowering (Kim *et al.*, 2013; Kang *et al.*, 2015). Similar results were found in the *hos15* mutant in inductive long-day (LD) conditions, where *AGAMOUS-LIKE 19* (*AGL19*) and *AGL24* as well as *CO* and *SOC1* in these conditions (Park *et al.*, 2019) were up-regulated.

Kim *et al.* (2013) did not observe altered expression or differential H3K9Ac or H3K27Ac levels of the flowering time regulator *FLOWERING LOCUS C* (*FLC*) under SD or LD conditions in *hda9* mutants. Also, Park *et al.* (2019) reported that levels of the floral repressor *FLC* were unchanged in the *hos15* mutant background in LD conditions. Kang *et al.* (2015), however, demonstrated that loss of *HDA9* led to a slight reduction in *FLC*, as well as *MAF4* and *MAF5*, mRNA levels in both LD and SD conditions. Yet, their genetic analyses suggested that HDA9 mediates flowering time largely independently of FLC (Kang *et al.*, 2015). However, a very recent report showed that HDA9 associates with the CURLY FLOWER (CLF)–PRC2 transcriptional repressor complex, to regulate *FLC* repression and thereby flowering time, based on a forward genetic approach (Zeng *et al.*, 2020). The authors reported that *FLC* transcription was markedly up-regulated in the *hda9* mutant background in LD conditions and accordingly, HDA9 associated with the *FLC* locus and directly mediated local histone deacetylation (Zeng *et al.*, 2020). CLF–PRC2 recruitment and H3K27Me3 levels at the *FLC* locus were partly reduced in the *hda9* mutant background. This suggests that HDA9-mediated H3K27 deacetylation is a prerequisite for CLF–PRC2-mediated repressive H3K27Me3 marker deposition and thereby *FLC* repression (Zeng *et al.*, 2020). Interestingly, genome-wide analysis showed that this requirement applies across the genome and is not restricted to *FLC* alone. In addition, HDA9 was shown to physically interact with the CLF–PRC2-interacting proteins VP1/ABI3-LIKE 1 (VAL1) and VAL2, that possess a plant-specific B3 DNA-binding domain and recognize the *CME* element in the *FLC* promoter. Hence, HDA9 acts in concert with the CLF–PRC2 complex to suppress the expression of *FLC* and the floral integrator *FLOWERING LOCUS T* (*FT*), via mutual physical interactions with the epigenome readers VAL1 and VAL2 (Zeng *et al.*, 2020).

Further evidence showed that a mutation in the *FT* locus suppressed the *hda9* early flowering phenotype, and *FT* mRNA levels were increased in the *hda9* mutant background (Kang *et al.*, 2015; Park *et al.*, 2019; Zeng *et al.*, 2020). This suggests that *HDA9* acts upstream of *FT* in flowering time regulation. This effect is likely to be a direct consequence of altered *AGL19* transcription in the *hda9* mutant, as H3Ac levels of the *FT* locus were unaltered in the *hda9* mutant background, in contrast to the *AGL19* locus (Kang *et al.*, 2015). Genetic analyses further indicated that HDA9 negatively regulates the autonomous flowering pathway, as the late-flowering phenotype of a plant line carrying an active *FRIGIDA* allele was partially suppressed by the *hda9* mutation (Kang *et al.*, 2015). The photoperiodic pathway was similarly affected by HDA9, although to a lesser extent. In LD conditions, double mutants between *hda9* and *gigantea* (*gi-2*) or *constans* (*co-101)* displayed a late flowering phenotype compared with the wild type; however, each double mutant flowered slightly earlier than the respective single mutants (Kang *et al.*, 2015). Similar results were presented by Park *et al.* (2019), who demonstrated that *HOS15* might function upstream of *GI*, *CO*, and *FT*, as the respective double mutant combinations with *hos15* were late flowering in LD conditions, whereas the *hos15* single mutants were early flowering. The latter effect is most probably due to the high levels of *GI* expression in *hos15* mutants due to H3 hyperacetylation at the *GI* locus (Park *et al.*, 2019). Furthermore, in the absence of *hos15*, the HDA9–HOS15–LUX/ELF3 complex cannot target the *GI* promoter for deacetylation. Notably, the early flowering of the *hos15* mutant under SD conditions was independent of GI (Park *et al.*, 2019).

Taken together, the role of HDA9 in flowering time control is highly complex as it depends on many environmental factors, including daylength, where HDA9 appears to modulate at the same time the expression of positive (e.g. *AGL19*, *GI*, and *FT*) and negative floral regulators (e.g. *FLC*). For example, the observation that *hda9* mutants flower like the wild type in LD conditions (Kang *et al.*, 2015; Zeng *et al.*, 2020), despite markedly high *FLC* repression levels in this mutant (Zeng *et al.*, 2020), can be possibly explained by the misexpression of other floral regulators such as *FT.*

The complex and sometimes contrasting findings in Arabidopsis prohibit drawing firm conclusions on the role of HDA9 in flowering as of yet. However, the role of HDA9 is at least partially conserved in different plant species, as the HDA9 homolog of the oil seed and vegetable crop *Brassica juncea* (*BjuHDA9*) was shown to interact with the promoters of *BjuSOC1* and *BjuAGL24* (Jiang *et al.*, 2018). Interestingly, *BjuHDA9* transcript levels were higher in an SD photoperiod than in LDs (Jiang *et al.*, 2018). Moreover, overexpression of the floral regulator *BjuAGL18* resulted in the transcriptional up-regulation of *BjuHDA9* during flowering (Yan *et al.*, 2018). Whether HDA9 is also transcriptionally regulated by the photoperiod and/or floral regulators in Arabidopsis remains to be investigated.

## HDA9 controls leaf aging, senescence, autophagy, and cellular proliferation and de-differentiation

Despite the delayed flowering initiation observed in *hda9* mutants, HDA9 is considered to play a generic role in promoting developmental progression (Suzuki *et al.*, 2018). This was proposed based on quantification of leaf heteroblasty progression of *hos15* mutants, which revealed a slightly delayed juvenile to adult phase transition, which probably also accounts for *hda9* (Suzuki *et al.*, 2018). In addition, HDA9 promotes cell proliferation in leaf primordia. Hence, *hda9* mutants produce smaller leaves with a reduced number of palisade cells (Suzuki *et al.*, 2018). In contrast, *HDA9* also promotes cellular de-differentiation (Lee *et al.*, 2016), as *hda9* mutants displayed reduced ability of pluripotent callus formation, and several genes involved in the de-differentiation process were down-regulated in leaves and calli of the *hda9* mutant. Moreover, *HDA9* itself is transcriptionally up-regulated in callus tissues (Lee *et al.*, 2016).

Compelling evidence for a role for HDA9 in developmental progression was provided by Chen *et al.* (2016), who demonstrated that HDA9 stimulates leaf aging and senescence by targeting multiple senescence-regulating pathways simultaneously. In a search for PWR-interacting proteins by immunoaffinity purification followed by MS, HDA9, WRKY53, and AHL22 were identified as the most abundant peptides co-purifying with PWR (Table 3). Subsequent analysis indicated that age-related and dark-induced leaf senescence was delayed in *hda9* and *pwr* single mutants and their *hda9 pwr* double mutant combination (Chen *et al.*, 2016; Yang *et al.*, 2020). Transcription of various positive regulators of senescence, such as *SENESCENCE 4* (*SEN4*), *SENESCENCE ASSOCIATED GENE 12* (*SAG12*), and *SAG113*, was attenuated in the *hda9* mutant background (Chen *et al.*, 2016). Similarly, down-regulation of a significant fraction of genes known to be repressed during senescence was impaired in the *hda9* mutant background (Chen *et al.*, 2016). In agreement with the influence of HDA9 on the senescence transcriptome, the protein was mildly up-regulated in early-senescent leaves. Abscisic acid (ABA)-responsive genes were significantly down-regulated in *hda9* mutants, suggesting that the ABA phytohormone signaling pathway, known to be involved in senescence (Jibran *et al.*, 2013), is impaired in these mutants (Chen *et al.*, 2016). Furthermore, among the senescence-associated genes differentially expressed in *hda9* is *WRKY57*, encoding a transcription factor involved in the repression of jasmonic acid (JA) during leaf senescence that was demonstrated to be a direct target of HDA9 (Chen *et al.*, 2016).

The observation that the W-box promoter element, recognized by WRKY transcription factors, was over-represented among HDA9 chromatin-binding targets also suggests a functional connection between HDA9 and WRK53 in senescence (Chen *et al.*, 2016). However, the role of HDA9–WRKY53 interactions in regulating leaf senescence remains to be confirmed empirically.

Autophagy is one of the processes involved in leaf senescence (Hanaoka *et al.*, 2002; Avila-Ospina *et al.*, 2014). Autophagy is a metabolic process in which cytoplasmic components such as proteins and dysfunctional organelles are sequestered to the vacuole or lysosome for degradation and recycling, which is important for tolerance to adverse environmental conditions. The process of autophagy is regulated by the so-called autophagy-related genes (*ATG*s).

A recent study demonstrated the involvement of HDA9 in the transcriptional regulation of *ATG*s (Yang *et al.*, 2020). The authors showed that nitrogen starvation and darkness induce autophagy and modulate *ATG* expression. Based on the premise of light-mediated transcriptional regulation of these *ATG*s, the versatile light signaling regulator bZIP transcription factor HY5 (Gangappa and Botto, 2016) was selected for further study. Indeed, HY5 negatively regulates autophagy in darkness and under nitrogen starvation conditions, and was shown to target the promoters of *ATG5* and *ATG8e* (Yang *et al.*, 2020). As a next step, HDA9 was identified in a screen for HDACs that interact with HY5 (Table 3), and mutants in *hda9* are more tolerant of nitrogen starvation than the corresponding wild type and displayed more autophagosomes. Accordingly, ATG5 and ATG8e transcript and protein levels were enhanced in the *hda9* mutant, and disruption of autophagy by mutating *atg5* or *atg7* abolished the enhanced nitrogen starvation tolerance phenotypes of *hy5* and *hda9* mutants. Accordingly, ChIP-PCR experiments indicated that HDA9 is targeted to the *ATG5* and *ATG8e* genomic loci in a HY5-dependent manner. Double mutant analysis confirmed that HY5 and HDA9 synergistically regulate cell autophagy upstream of ATGs by H3K9 and H3K27 deacetylation of the *ATG5* and *ATG8e* genomic loci, thereby regulating their expression (Yang *et al.*, 2020). Interestingly, the HY5–HDA9 complex dissociated from the chromatin of *ATG5* and *ATG8e* in response to darkness and nitrogen starvation, and the HY5–HDA9 protein–protein interaction was broken. In addition, darkness and nitrogen starvation conditions led to HY5, but not HDA9, 26S proteasomal degradation in a COP1-dependent manner (Yang *et al.*, 2020).

Taken together, a model was proposed whereby, under light and high nitrogen conditions, HY5 recruits HDA9 to repress *ATG* expression by decreasing acetylation levels, thereby suppressing cell autophagy. In response to nitrogen starvation and darkness, HY5 is degraded in a COP1-dependent manner, leading to the dissociation of HDA9 and acetylation of *ATG*s, followed by their transcriptional induction and activation of cell autophagy, which ultimately results in enhanced tolerance to these environmental conditions (Yang *et al.*, 2020).

## The role of HDA9 in regulating seed dormancy and germination

Seed dormancy is defined as a state of quiescence in viable seeds, during which germination is prohibited, even if environmental conditions are favorable for germination (e.g. seasonal optimal temperature, moisture, and light conditions; Baskin and Baskin, 2004; Née *et al.*, 2017). Treatment of dormant Arabidopsis wild-type Columbia-0 (Col-0) seeds with the HDAC inhibitors trichostatin-A (TSA) and butyric acid sodium salt released dormancy in a dose-dependent manner. Subsequent reverse genetic analysis revealed that mutants in *hda9* displayed reduced dormancy (Van Zanten *et al.*, 2014). Moreover, *hda9* mutants germinated faster (Van Zanten *et al.*, 2014; Baek *et al.*, 2020) and exhibited improved seed longevity (storability) (Van Zanten *et al.*, 2014). The role of different HDACs in seed biology, however, depends on the species studied (Van Zanten *et al.*, 2013). For instance, TSA application leads to a delay in germination in maize (Zhang *et al.*, 2011).

Germination and dormancy are tightly regulated by the balance between the phytohormones gibberellin (GA) and ABA, where GA typically stimulates germination and ABA is associated with the repression of germination and dormancy enhancement (Finkelstein *et al.*, 2008). ABA levels were reduced in seeds of *hda9* mutants and increased in heterotrophic seedlings (Baek *et al.* 2020). It remains an open question if and how the recently identified interaction between HDA9, ABI3, and ABI4 (Baek *et al* 2020; Khan *et al.*, 2020) contributes to regulating seed dormancy and germination. However, pharmacological analysis indicated that ABA and GA sensitivity of seeds was unaltered in the *hda9* mutant (Van Zanten *et al.*, 2014), suggesting that HDA9 affects dormancy and germination largely independently of these phytohormones. Accordingly, meta-analysis of transcriptome data obtained from wild-type and *hda9* mutant seeds, compared with published datasets, did not reveal a significant similarity that would suggest an involvement of GA and ABA (Van Zanten *et al.*, 2014). However, unexpectedly, many of the differentially regulated genes in the *hda9* mutant coded for factors involved in photosynthesis, the Calvin cycle, and secondary metabolism (Van Zanten *et al.*, 2014). This included the 2B subunit of Rubisco and Rubisco activase (RCA). ChIP-PCR experiments confirmed that H3K9Ac levels on the loci of these genes were increased in *hda9* compared with the wild type, especially in the 5' (+500 bp) region (Kim *et al.*, 2013; Van Zanten *et al.*, 2014). Moreover, Rubisco protein levels were enhanced in *hda9* mutant dry seeds (Van Zanten *et al.*, 2014). Taken together, HDA9 can be considered a positive regulator of seed dormancy and a repressor of germination and of vegetative properties in dry seeds. Interestingly, the opposite function was shown for the HDA9 homologs HDA6 and HDA19—these HDACs are involved in repression of embryonic properties in autotrophic seedlings (Tanaka *et al.*, 2008).

ASG2 (ALTERED SEED GERMINATION 2) is a WD40 and Tetratrico Peptide Repeat (TPR) domain protein that is involved in ABA signaling. Mutant *asg2* seeds exhibited increased weight, oil body density, and higher fatty acid contents that affected seed germination (Dutilleul *et al.*, 2016; Ducos *et al.*, 2017). The farnesylated form of ASG2 was shown to interact with HDA9 in the cytosol, but not in the nucleus (Ducos *et al.*, 2017) (Fig. 1; Table 3). Future work should address the biological function of this interaction, especially whether HDA9 affects seed fatty acid content and how it is connected through ASG2 to the diverse roles of HDA9 in seed dormancy, germination, repression of vegetative properties, and possibly other biological processes.

## Involvement of HDA9 in regulating responses to environmental signals: drought and salt stress

Plants have to deal with a large number of biotic and abiotic cues (Zhu, 2016), and HDA9 has been reported to play a role in orchestrating the responses to various environmental conditions (Zheng *et al.*, 2016; Tasset *et al.*, 2018; Shen *et al.*, 2019; van der Woude *et al.*, 2019; Yang *et al.*, 2019; Zheng *et al.*, 2020) (Fig. 1; Table 2). For instance, *hda9* mutants accumulate high levels of iron in their roots, suggesting a role in iron homeostasis (Xing *et al.*, 2015) and, as described above, HDA9 contributes to regulating darkness- and nitrogen starvation-mediated autophagy/leaf senescence (Yang *et al.*, 2020). In addition, HDA9 is reported to function as a negative regulator of salt and drought stress tolerance, due to its repressive effect on stress-responsive genes in Arabidopsis (Zheng *et al.*, 2016, 2020). Observations in broccoli (*Brassica oleracea*) suggest that salt-mediated regulation of *HDA9* transcript levels may have a role in bud senescence (Yan, *et al.*, 2020). Arabidopsis *hda9* mutants displayed a decrease in the inhibition of seed germination and root growth, and thus an increase in tolerance to high NaCl concentration and simulated drought stress (PEG; polyethene glycol) conditions compared with the wild type (Zheng *et al.*, 2016, 2020). In two recent studies, Baek *et al.* (2020) and Khan *et al.* (2020), however, proposed that HDA9 and PWR are positive regulators of physiological drought stress tolerance (i.e. progressive drought by withholding watering). Mutants in *pwr* (Khan *et al.*, 2020) and *hda9* (Baek *et al.*, 2020) displayed reduced sensitivity to ABA regarding stomatal closure, and *HDA9* was transcriptionally induced under drought conditions (Baek *et al.*, 2020). Interestingly, yeast two-hybrid and Co-IP analyses demonstrated that HDA9 physically interacts with the transcription factors ABI4 (Baek *et al* 2020; Khan *et al.*, 2020) and ABI3 (Khan *et al.*, 2020) (Fig. 1; Table 3). Combined, the data support a model in which a PWR–HDA9–ABI4 complex targets the loci of ABA catabolism and ABA signaling genes and regulates their histone acetylation status and transcription (Baek *et al.*, 2020; Khan *et al.*, 2020). Transcript levels of the ABA catabolism genes *CYP707A1* (*hda9* and *pwr*) and *CYP707A2* (*hda9*) were indeed enhanced, whereas ABA phytohormone levels were reduced in *hda9* and *abi4* mutant plants under drought stress. Moreover, H3 acetylation levels were enhanced at the *CYP707A1* locus in the *hda9* and *pwr* mutant backgrounds (Khan *et al.*, 2020). Stomatal aperture and water loss were accordingly increased in these mutant backgrounds, resulting ultimately in dehydration hypersensitivity (Baek *et al.*, 2020; Khan *et al.*, 2020).

Similar to *CYP707A1* and *CYP707A2* (Baek *et al.*, 2020; Khan *et al.*, 2020), Zheng *et al.* (2016, 2020) found that several drought stress-related genes were highly induced in the *hda9* mutant background upon drought/salt stress application, which correlated with enhanced H3K9Ac levels in promoter regions of a selection of these genes. Furthermore, yeast two-hybrid analysis indicated an interaction of HDA9 with HDA6, HDA19, and AtCYP1-1 (cyclophilin-like peptidyl-prolyl *cis–trans* isomerase family protein) (Table 3), all of which have been associated with salt and/or drought stress before (Zheng *et al.*, 2016). However, later work from Yuan *et al.* (2019) did not confirm an interaction between HDA6, or HDA19, and HDA9, and the possible association between HDA9 and HDA6, HDA19 and AtCYP1-1 in drought and/or salt stress responsiveness was not functionally validated *in planta* (Zheng *et al.*, 2016).

The interaction between WRK53 and HDA9, that was previously described in the context of leaf senescence (Chen *et al.*, 2016), was confirmed (Zheng *et al.*, 2020). In contrast to HDA9, in the latter study WRKY53 was shown to act as a positive regulator of salt and drought stress responses, and the mutual and antagonistic roles of HDA9 and WRKY53 have now been elucidated in great molecular depth (Zheng *et al.*, 2020). In detail, the authors showed that HDA9 repressed *WRKY53* transcription—and therewith several *WRKY53* target genes—under non-stressed conditions and thereby prevented *WRKY53* gene induction under salt stress. Unexpectedly, HDA9 did not, however, associate with the chromatin of *WRKY53* target genes, nor were histone acetylation levels at the *WRKY53* locus affected in the *hda9* mutant. However, H3K4Me2/Me3 levels were enhanced, correlating with the increased *WRKY53* expression in the *hda9* mutant background under salt stress (Zheng *et al.*, 2020). These observations prompted the authors to test whether HDA9 could target the WRKY53 protein directly. Indeed, post-translational K12Ac, K26Ac, K27AC, K58Ac, K169Ac, K175Ac, and K268Ac modification levels of the WRKY53 protein were higher in the *hda9* mutant and lower in a *HDA9* overexpression line, which was confirmed by several biochemical validations (Zheng *et al.*, 2020). The authors thus revealed that HDA9 is able to modify the acetylation status of a non-histone protein.

Additional studies indicated that HDA9 represses WRKY53 *cis* transcriptional activity by preventing the transcription factor from binding to its own promoter (Zheng *et al.*, 2020). Accordingly, the central deacetylase domain of HDA9 interacts directly with the WRKY53 DNA-binding domain. HDAC inhibition with TSA did not interfere with the negative effect of HDA9 on WRKY53 DNA binding capacity, suggesting that this probably occurs independently of WRKY53 lysine deacetylation. WRKY53 lysine acetylation is, however, important for WRKY53 transcriptional activity *in trans* (Zheng *et al.*, 2020).

Interestingly, H3K9Ac/H3K27Ac levels were increased and decreased, respectively, in WRKY53 overexpression and *wrky53* mutant lines, suggesting that WRKY53 in turn regulates HDA9 activity. This was confirmed by direct HDAC activity assays using purified HDA9 protein, derived from the *WRKY53* overexpression and *wrky53* mutant line, and by experiments with recombinant WRKY53 protein. The repression of HDA9 activity required the WRKY53 DNA-binding domain, which probably masks the HDAC catalytic domain (Zheng *et al.*, 2020).

In conclusion, HDA9 modulates salt and drought stress tolerance responses by directly targeting and repressing the DNA binding and transcriptional activity of the high hierarchical positive regulator of stress responses; WRKY53 (Zheng *et al.*, 2020).

## Involvement of HDA9 in regulating responses to environmental signals: thermomorphogenesis

While HDA9 is considered to function mainly as a negative regulator of salt and drought stress responsiveness, the protein was identified as a positive regulator of plant thermomorphogenesis—a suite of architectural traits induced by plants to mitigate negative effects of mildly increased temperatures—by improving cooling capacity (Quint *et al.*, 2016; Casal and Balasubramanian, 2019). Thermomorphogenesis is mediated by the high temperature-induced transcription factor PHYTOCHROME INTERACTING FACTOR4 (PIF4) (Koini *et al.*, 2009; Sun *et al.*, 2012) and regulated by the EC component ELF3 (Box *et al.*, 2015; Raschke *et al.*, 2015). PIF4 activates the expression of auxin biosynthesis genes, including that encoding the rate-limiting enzyme YUCCA8 (YUC8), that subsequently stimulates auxin accumulation required for inducing thermomorphogenesis (Franklin *et al.*, 2011; Sun *et al.*, 2012), in concert with the brassinosteroid phytohormones (Ibañez *et al.*, 2018). Furthermore, high temperatures lead to the eviction of the histone variant H2A.Z-containing nucleosomes from promoters of thermo-responsive genes, which then allows for the binding of transcriptional regulators, including PIF4, to the DNA (Kumar and Wigge, 2010; Cortijo *et al.*, 2017).

Mutants in HDA9 and PWR are impaired in thermomorphogenesis, as exhibited by traits such as reduced hypocotyl elongation and maintenance of a compact rosette (Tasset *et al.*, 2018; Shen *et al.*, 2019; van der Woude *et al.*, 2019). Some warm temperature-mediated features were, however, retained in *hda9*. For instance, the expression of *HEAT SHOCK PROTEIN 70* (*HSP70*), a warm temperature-induced marker gene (Kumar and Wigge, 2010), and high temperature-induced flowering were comparable between *hda9* and wild-type plants. This contrasted with *pwr* mutants that displayed reduced *HSP70* expression and reduced sensitivity of thermal floral induction (Tasset *et al.*, 2018). Moreover, opposite to PWR (Tasset *et al.*, 2018), HDA9 is not involved in regulating *PIF4* at the transcriptional level under warm temperatures (van der Woude *et al.*, 2019). Interestingly, unlike *pif4* mutants, *hda9* loss-of-function alleles retain their responsiveness to light signals that induce the shade avoidance response that resembles thermomorphogenesis and is considered to be a competitive response to outgrow shading in dense canopies (Ballaré and Pierik, 2017). Furthermore, the effects of HDA9 on thermomorphogenesis occurred independent of the light and temperature sensor phytochrome B (phyB) (van der Woude *et al.*, 2019). Together, this suggests that HDA9 is part of a thermosignaling pathway that operates independently of shade avoidance and temperature-induced flowering regulation.

At the protein level, HDA9 accumulates at dawn and becomes less abundant over the photoperiod in response to high temperature (27 °C), whereas no marked (diurnal/circadian) changes in HDA9 protein contents were observed at control temperatures. HDA9 mRNA and protein were mainly detected in young seedlings shortly after germination and declined during seedling establishment. Together, this suggests that HDA9 protein is regulated by temperature cues and can be considered as an early regulator of thermomorphogenesis (van der Woude *et al.*, 2019).

Gene Ontology enrichment analysis revealed that high temperature-induced up-regulation of auxin-related genes was impaired in *hda9* mutants (van der Woude *et al.*, 2019), and subsequent analysis confirmed that this included *YUC8* (Sun *et al.*, 2012). In agreement, warm temperature-induced *YUC8* induction was impaired in *pwr* mutants as well (Tasset *et al.*, 2018). In line with reduced *YUC8* expression, bioactive auxin (indole-3-acetic acid; IAA) levels were low in the *hda9* mutant under warm temperature conditions, whilst the YUC8 enzyme substrate indole-3-pyruvic acid (IPyA) accumulated to high levels (van der Woude *et al.*, 2019). ChIP-PCR analyses revealed hyperacetylation of the *YUC8* promoter in the *hda9* and *pwr* mutant backgrounds under high temperature and also in control temperature conditions for *pwr*, suggesting that histone deacetylation is required for *YUC8* expression. Interestingly, HDA9-mediated H3K9K14 deacetylation of nucleosomes was associated with low H2A.Z levels at warm temperatures at the *YUC8* locus, whereas *hda9* mutants displayed high H2A.Z levels. These high H2A.Z levels consequently led to reduced PIF4 binding capacity to the G-box promoter element, which explains attenuated *YUC8* transcriptional induction, prohibition of auxin biosynthesis, and suppression of thermomorphogenesis, in the *hda9* mutant background. Probably, PWR is involved in this as well, as genes misregulated in *pwr* mutants exhibited significant overlap with known H2A.Z-enriched genes, and with differentially expressed genes in mutants disturbed in H2A.Z deposition (Tasset *et al.*, 2018).

Altogether, HDA9–PWR-mediated deacetylation is associated with thermomorphogenesis via an induction of gene transcription (of *YUC8*), by promoting net depletion of the repressive histone variant H2A.Z. The role of *PWR* in thermomorphogenesis regulation appears broader than that of HDA9, given the more pleiotropic phenotypes of *pwr* compared with *hda9*. Whether HOS15 plays an active role in regulating thermomorphogenesis as well could be addressed in future studies.

It is worth mentioning that the notable role of HDA9 in activating gene expression is atypical, since HDACs are generally considered to act as transcriptional co-repressors (Li *et al.*, 2002; Tian *et al.*, 2005; Perrella *et al.*, 2013). Further studies are required to reveal if HDA9 has a similar transcriptional activating role in other HDA9-mediated processes. Recent reports using genome-wide HDA9 ChIP-sequencing surveys showed that HDA9 indeed associates mainly with actively transcribed genes and that HDA9 binding positively correlates with gene expression (Kang *et al.*, 2015; Chen *et al.*, 2016; Kim *et al.*, 2016; Mayer *et al.*, 2019).

## Involvement of HDA9 in plant immunity

In general, plants display two distinct types of immunity: pattern-triggered immunity (PTI) and effector-triggered immunity (ETI) to defend against microbial pathogens. PTI is based on recognition of conserved microbial or pathogen-associated molecular patterns (MAMPs and PAMPs), whereas ETI is based on recognition of pathogen-associated effectors or toxins (Jones and Dangl, 2006; Miller *et al.*, 2017). Many of these pathogen-associated effectors are recognized by nucleotide-binding leucine-rich repeat/NOD-like intracellular immune receptor (NB-LRR or NLR) proteins (Meyers *et al.*, 2003). Tight regulation of *NLR* genes is vital to balanced plant growth and defense. Constitutive expression of *NLR* genes suppresses plant growth and causes autoimmunity, whereas, on the other hand, adequate induction of *NLR* gene expression is crucial for timely recognition of pathogens and effective defense initiation.

Recent evidence indicated important roles for HDA9 and HOS15 in *NLR* transcriptional regulation (Yang *et al.*, 2019). Arabidopsis plants defective in *HDA9* and *HOS15* show enhanced resistance against *Pseudomonas syringae* pv*. tomato* DC3000 (Yang *et al.*, 2019). However, neither *HOS15*, *HDA9* transcript, nor protein levels were altered in response to pathogen infection. Similarly, neither HDA9 nuclear–cytoplasmic transport nor HOS15–HDA9 protein–protein interaction was affected.

Nevertheless, *hda9* and *hos15* mutants together regulate a large fraction (approximately one-third) of known *NLR* genes in the genome (Yang *et al.*, 2019). ChiP-seq experiments indicated that HDA9 and HOS15 target largely the same subset of *NLR* genes, and mainly those that are differentially regulated at the transcriptional level in the *hos15* mutant background compared with the wild type. However, unlike in the *hos15* mutant, not many defense response genes were differentially regulated in the *hda9* mutant in the absence of infection. This indicates that HDA9 requires a pathogenic trigger for its involvement in defense regulation. Indeed, H3K9Ac status of a selection of NLR genes was only enhanced in the *hda9* mutant background upon pathogen infection, whereas acetylation levels of these loci were constitutively high in the *hos15* mutant background (Yang *et al.*, 2019).

How infection is able to activate HDA9-mediated defense remains unknown. Post-translational modifications triggered by infection of inactive HDA9 that is potentially already bound to its target loci may play a role. In addition, WRKY DNA-binding proteins might be responsible for recruiting HDA9 (and possibly HOS15) to its *tailLR* gene target loci once plants are infected, as W-boxes are the only known *cis*-elements that are present in NLR promoter regions. This could point to a possible role for WRKY53 in HDA9-mediated *NLR* expression regulation (Chen *et al.*, 2016; Yang *et al.*, 2019; Zheng *et al.*, 2020). Testing this hypothesis would require further studies.

Taken together, HDA9 and HOS15 function in the same pathway to suppress immunity. Given the constitutively enhanced immunity status of *hos15* mutants, the typical stunted rosette phenotype of *hos15* and possibly also *hda9* mutants could be interpreted as a mild autoimmune phenotype; that is, the growth–immunity trade-off in these mutants has possibly shifted towards immunity at the expense of growth, despite *NLR* genes not being induced in *hda9* in non-infected conditions.

## HDA9 in the larger HDAC context

Despite the fact that HDA9 directly controls many physiological and molecular traits governing plant development, growth, and responses to a changing environment (Fig. 1), it is unlikely that HDA9 operates in isolation independent of other HDAC proteins. Evidence suggests that HDA9 can act in parallel, redundantly, synergistically, or antagonistically to other members of the HDAC family. For instance, *hda9* mutants display typical blunt and bulged siliques (tips) attributed to enhanced valve cell elongation (Yuan *et al.*, 2019). This phenotype was not observed in *hda6* single mutants. However, the *hda9 hda6* double mutant showed additively exaggerated bulged silique and valve cell elongation phenotypes, suggesting that HDA6 and HDA9 redundantly control silique morphology (Yuan *et al.*, 2019). These phenotypes emerge through the coordinated regulation of auxin signaling genes by HDA6 and HDA9, as many auxin-related genes and auxin signaling are additively affected in the single and double mutants (Yuan *et al.*, 2019).

On the contrary, different HDACs may also act independently by targeting specific branches of regulatory molecular networks that either translate the same input to diverse phenotypic outcomes or translate different input to the same phenotypic outcomes. For instance, in the context of thermomorphogenesis, HDA9 has distinct and overlapping functions with HDA15 and HDA19. Mutants of *HDA9* and *HDA19* showed impaired warm temperature-induced hypocotyl elongation, while, on the contrary, a mutant of *HDA15* exhibited a constitutive enhanced thermomorphogenesis response (Shen *et al.*, 2019). This was reflected at the molecular level, as in *hda9* regulation of many warm temperature-regulated genes was impaired, while in *hda15* many warm temperature response genes are differentially regulated already at control temperature conditions. In the *hda19* mutant, mostly stress-regulated genes were affected, at both control and high temperature conditions (Shen *et al.*, 2019). Thus, these HDACs target distinct sets of genes and have distinct functions in the regulation of plant thermomorphogenesis. At the same time, a large fraction of misregulated genes involved in metabolism were shared between *hda9* and *hda15*, suggesting that these HDACs may control the same metabolic pathways, but diverge in the regulation of thermomorphogenesis (Shen *et al.*, 2019). Yet, HDA9 may have antagonistic roles with respect to other HDACs. An example of this is the aforementioned role of HDA9 in repressing vegetative traits in seeds (Van Zanten *et al.*, 2014), whereas HDA6 and HDA19 together repress embryonic properties in autotrophic young seedlings (Tanaka *et al.*, 2008).

## Concluding remarks

Diverse roles of HDA9 in the regulation of a multitude of plant traits and responses to the environment have been described in recent years (Fig. 1; Table 2) in concert with few established direct interacting proteins (Fig. 1; Table 3). Nevertheless, several important questions remain to be answered. For instance, it is currently unclear if the cytosolic HDA9 population functions in the deacetylation of non-histone proteins other than WRKY53 (Zheng *et al.*, 2020) and whether the atypical role of HDA9 as a conditional activator of gene transcription extends beyond *YUC8* (Van der Woude *et al.*, 2019). Another intriguing question is why *hda9* mutants were hardly identified in reverse genetic mutant screens, despite its pleiotropic roles in diverse plant processes. Furthermore, future efforts could address how knowledge on Arabidopsis HDA9 can be utilized and translated to improve crop performance and yield in response to climate change.

As described in detail in this review, HDA9 has many faces, as its mode of action is tailored to specific trait/response and sometimes has apparent opposite effects (as seen for drought stress tolerance for instance) (Table 2; Fig. 1). Nevertheless, the involvement of HDA9 in regulating responsiveness to diverse environmental stimuli (e.g. pathogens, salt, drought, high temperature, darkness, and iron) on one hand, and diverse plant responses to these stimuli (e.g. growth acclimation, autophagy, senescence, aging, dormancy, and germination) on the other, suggests that HDA9 is an essential player in the molecular networks mediating optimal plant performance under suboptimal environmental conditions.

It is likely that many more unidentified HDA9-mediated phenotypes and interacting proteins remain to be discovered. HDA9 is able to physically interact with several transcription factors (e.g. WRKY53, HY5, ABI3, and ABI4) (Fig. 1; Table 3), which might contribute to establishment of HDA9-dependent epigenetic states, particularly in response to environmental stimuli. In this regard, it has been extensively demonstrated that transcription factors can directly recruit histone modifiers to their DNA targets to reinforce the local epigenetic landscape (Bonasio *et al.*, 2010). Given the substantial difference in HDA9-interacting proteins compared with those identified for HDA6 and HDA19 on one hand (Perrella *et al.*, 2013; Mehdi *et al.*, 2016; Ning *et al.*, 2019), and the positive correlation of HDA9 presence with gene expression (Kang *et al.*, 2015; Chen *et al.*, 2016; Kim *et al.*, 2016; Mayer *et al.*, 2019; van der Woude *et al.*, 2019) on the other, we speculate that HDA9 may be fundamentally divergent from related HDACs.

Intriguingly, *hda9* mutants also display impaired histone methylation and miRNA levels (Kim *et al.*, 2009), suggesting a possible crosstalk with other epigenetic modifications. Similar mechanisms have been shown for other HDACs, including HDA6 that regulates flowering time through the association with the histone demethylase FLOWERING LOCUS D (FLD) (Yu *et al.*, 2011). Furthermore, HDA6 interacts with the DNA methyltransferase MET1, thereby regulating cytosine methylation and rDNA loci in heterochromatic regions (To *et al.*, 2011; Liu *et al.*, 2012). However, how HDA9 acts in concert with other HDACs to mediate PRC2-dependent histone trimethylation and whether such a mechanism can occur on other loci rather than *FLC* requires further investigation. Similarly, the involvement of HDA9 in regulating miRNA genesis is not yet fully understood.

To date, ‘HDA9’ as a search input in the NCBI PubMed database (2 July 2020; (https://www.ncbi.nlm.nih.gov/pubmed/?term=HDA9) recovered 25 papers out of which 22 were published after 2016 and no less than 15 in 2019/2020. Thus, our knowledge on this previous undercharacterized protein is currently accumulating rapidly, and integration and cross-validation of findings is needed to fully appreciate the impact that HDA9 has on plant growth and development, and environmental responses. This review discussing the multiple functions of HDA9 aims to help the growing HDA9 community in achieving this goal.
